# Human health risks posed by exposure to PM10 for four life stages in a low socio-economic community in South Africa

**DOI:** 10.11604/pamj.2014.18.206.3393

**Published:** 2014-07-07

**Authors:** Nomsa Duduzile Lina Thabethe, Jacobus Christoffel Engelbrecht, Caradee Yael Wright, Maria Aletta Oosthuizen

**Affiliations:** 1 Tshwane University of Technology, Faculty of Science, Department of Environmental Health, Private Bag X680, Pretoria. 0001. South Africa; 2 Council for Scientific and Industrial Research, Climate Studies, Modelling and Environmental Health, P.O Box 395, Pretoria, 0001. South Africa

**Keywords:** Air pollution, exposure, human health risk assessment, community

## Abstract

**Introduction:**

Mine ash dumps, industries and domestic fuel use have a great impact on air quality and PM_10_ (particles with a diameter equal to or less than 10 μm) is a pollutant of particular concern.

**Methods:**

The objective of this study was to assess the human health risks posed by exposure to PM_10_ among a low socio-economic community. The Human Health Risk Assessment (HHRA) framework (i.e. hazard assessment, dose-response assessment, exposure assessment and risk characterization) was applied. PM_10_ concentrations were monitored for one month during winter and summer, respectively. A HHRA was conducted to assess whether the community was exposed to PM10 concentrations that may pose carcinogenic and non-carcinogenic health risks.

**Results:**

Generally, the residents were exposed to higher concentrations of PM_10_ during winter than summer, resulting in a higher risk to health during winter. Results of the HHRA showed that infants were exposed to a higher dose of PM10 than the other life stages when exposed to the same concentration due to differences in inhalation rates and the ratio between inhalation and body weight. However, they were at the same risk of developing adverse effects from exposure to the same concentration of PM_10_ as the other life stages were exposed to, because the ‘safe’ dose was also higher for infants and since all life stages, in general, are similarly affected by PM unless the chemical composition of the PM is known.

**Conclusion:**

This study recommends that infants and children, in particular, should not be exposed to air pollution from domestic fuel burning as one positive step to try and reduce their dose.

## Introduction

Particulate matter (PM) is a complex, heterogeneous mixture of smoke, soot, dust, salt, acids, and metals and varies in concentration, size, chemical composition, surface area and sources of origin [1]. PM may comprise, among others, sulphate, nitrate, ammonia, chloride and organic carbon particles [2]. PM_10_ is defined as particles with a diameter of equal to or less than 10 microns. Exposure to _10_ in particular, poses a definite risk to health because it is more likely to be inhaled and the fine fraction of PM_10_ (i.e. PM_2.5_) is respirable and may reach the alveolar region of the lung. Ultra-fine PM (i.e. PM_0.1_) that is inhaled settles in the lung by Brownian motion and some may be exhaled before it deposits in the lungs [3].

One area that is surrounded by industries, power stations and mines, all of which are recognised emitters of PM, is that of eMbalenhle, a township located in the Mpumalanga Province of South Africa, about 12 km from the town of Secunda. Domestic fuel burning has a great impact on air quality in eMbalenhle. The major sources of PM in eMbalenhle are motor vehicles, domestic fuel burning, mine ash dumps, dust from unpaved roads, landfills, agriculture, wildfires, waste burning, industrial sources and windblown dust from open lands. Given these air pollution sources, it was deemed necessary to estimate the likely human health risks posed by PM_10_ to the residents of eMbalenhle, an activity never done before for this geographic region, for future planning and management of air pollution in the area.

## Methods

The study comprised two parts namely an ambient air quality monitoring and a Human Health Risk Assessment (HHRA).

### Ambient air quality monitoring

A TOPAS airborne particulate monitor (Sira MC 090158/00) was used to measure the ambient PM_10_ concentrations to which the population of eMbalenhle were exposed. The monitor was installed at eMbalenhle Sasol Club, alongside the Department of Environmental Affairs (DEA) monitoring station (GPS coordinates: S26°33,039’ and E29°04,747’). Ambient PM_10_ concentrations were monitored for one month during winter (August 2010) and one month during summer (February 2011). DEA monitoring station data were also extracted for the same periods as the TOPAS instrument was installed for comparison purposes.

The particulate monitor was calibrated and maintained according to the manufacturers specifications. The fibre filters were replaced once after two weeks. Monitored data used to assess the health risks posed by exposure to PM_10_ in the population of eMbalenhle were compared with the South African National Ambient Air Quality Standard (NAAQS) [4]. The PM_10_ concentrations were measured in 15 minutes intervals, the raw data output was divided by a calibration factor of 2 and the 24-hour averages were calculated. The monitored PM_10_ concentrations were compared with the NAAQS which were originally set to protect human health.

### Human Health Risk Assessment

HHRA is a useful tool to estimate human health risks posed by exposure to a given environmental pollutant. HHRA have been applied in previous studies in South Africa, for example to estimate kerosene [5] and sulphur dioxide-related health risks [6]. However, a HHRA on PM_10_ has never been conducted in eMbalenhle. The HHRA framework applied in this study comprised four parts: hazard identification, exposure assessment, dose-response assessment and risk characterisation. The formal identification of PM_10_ as a hazard as well as the types of health risks that may occur as a result of exposure to PM_10_ was done from existing literature.

Dose-response assessment, i.e. how an individual will react to a particular exposure, was not performed in this study as the extent of the work requires comprehensive screening and additional health data presently not available in South Africa. Instead, the measured levels of PM_10_ were compared with the NAAQS. This national standard was therefore used as a benchmark value.

The information obtained during the hazard identification and the exposure assessment was used to estimate the concentrations of PM_10_ that are likely to cause significant health risks in humans. The PM_10_ monitored data were used to estimate how the different levels of exposure to PM_10_ can impact on the likelihood and severity of health effects.

It was postulated that the population of eMbalenhle was exposed to levels exceeding the NAAQS for PM_10_ that may have a negative impact on their health. It was assumed that inhalation was the most important route of exposure (not ingestion or dermal contact) and that people were exposed for 24 hours per day.

Two equations were used to characterise the risks posed by exposure to PM_10_, namely; the United States Environmental Protection Agency (USEPA) Exposure Factors Handbook and the EPA Integrated Risk Information System (IRIS) equations [7]. The magnitude, frequency and duration of exposure of the population of eMbalenhle to PM_10_ were unknown, thus the default values based on the USEPA equations were used. The South African 24-hour PM_10_ NAAQS of 120 μg/m3 was used as a benchmark value.

The USEPA equation was used to calculate the Field Average Daily Dose (FADD). In order to calculate the FADD, the average concentrations (C) of P PM_10_ monitored in eMbalenhle in August 2010 and February 2011 were multiplied by the Inhalation Rate (IR), Exposure Frequency (EF) and Exposure Duration (ED), and then divided by the Body Weight (BW) multiplied by the Average Time (AT).

FADD was calculated using the following equation:

1 FADD=C×IR×EF×ED/BW×AT

Where:

FADD is the dose the population of eMbalenhle may be exposed to when inhaling PM_10_ concentrations measured at eMbalenhle in August 2010 and February 2011, expressed in μg/kg/day.

C is the average value of the PM10 concentration in the atmosphere expressed in μg/m^3^. IR is the amount of contaminated medium (air) inhaled per unit time or event. It is expressed in m3/day.

EF (Exposure Frequency) which is 350 days, because it was assumed that a person will leave the area for about two weeks per year.

ED (Exposure Duration) expressed in years. For non-carcinogens assumed to be one year. BW is the average body weight of the receptor over the exposure period (kg). AT is the period over which exposure is averaged (1 year = 365 days). For non- carcinogens the AT equals ED (years) multiplied by 365 days [7].

The long-term inhalation rates for adults and children (including infants) were presented as daily rates (m^3^/day). It was assumed that the 95^th^ percentile inhalation rates for long-term exposures for infants, children and adults (males and females combined, unadjusted for body weight) range from 9.2 m^3^/day for infants from birth to 1 year, 16.6 m^3^/day for children aged 6 to 10 years to 21.4 m^3^/day for adults aged 31 to 40 years [7].

The Safe Average Daily Dose (SADD) was calculated as follows:

2 SADD=C×IR×ED/BW×AT

Where:

SADD is the dose that the population of eMbalenhle may be exposed to without suffering negative health risks, expressed in μg/kg/day. In this case the concentration C represents the South African 24-h standard for PM_10_ expressed in μg/m^3^. The rest of the formula is the same as described above.

The risks caused by exposure to PM_10_ in the population of eMbalenhle were characterised in terms of the potential risk to illness or symptoms in the exposed population. The information developed in the previous three steps (hazard identification, exposure assessment and dose-response assessment) was brought together in the risk characterisation step to quantify the potential health risks in the exposed population, expressed as a Hazard Quotient (HQ).

The HQ was calculated using the following equation [7]:

3 HQ=FADD/SADD

Where:

HQ is the Hazard Quotient (which is always unit less)

FADD is the Field Average Daily Dose calculated (in μg/kg/day)

SADD is the “safe” average daily dose calculated (in μg/kg/day)

Guidelines for interpreting HQ calculations are (Lemly, 1996):

HQ <0.1: no hazard exists;

HQ 0.1-1.0: the hazard is low;

HQ 1.1-10: the hazard is moderate; and

HQ >10: hazard is high

All statistical analyses were performed in Microsoft Excel. Ethical clearance was obtained for this study from the Tshwane University of Technology Research Ethics Committee on the 14^th^ March 2011 (Reference number: 2011/03/007).

## Results

### Measured results of PM_10_

**Figure 1:**
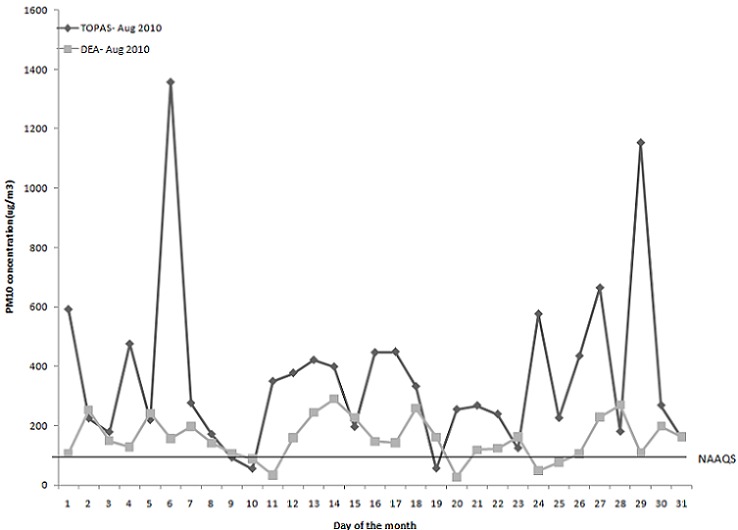
24-hour PM10 averages monitored with the TOPAS Monitor and by the DEA monitoring station in winter (August 2010)

**Figure 2:**
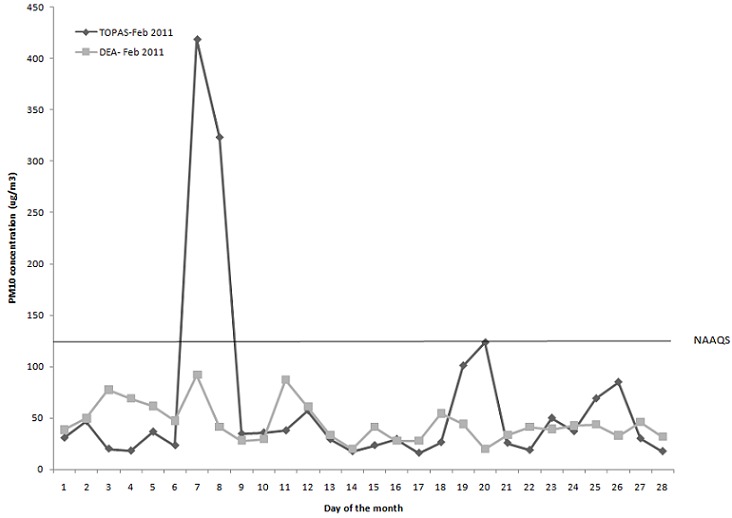
24-hour PM10 averages monitored with the TOPAS monitor and by DEA monitoring station in summer (February 2011)

The measured 24-hour average PM_10_ concentrations for winter (August 2010) and summer (February 2011) using both the TOPAS and DEA instruments are presented in Figure 1 and Figure 2, respectively. The PM_10_ concentrations measured in August 2010 were generally higher than the PM_10_ concentrations measured in February 2011. The 24-h average level for PM_10_ (August 2010) was 157.37 μg/m3 and the 24-h average NAAQS of 120 μg/m^3^ was exceeded on most of the days.

Figure 2 illustrates the PM_10_ concentrations monitored with the TOPAS and DEA instruments during February 2011. The particle pollution reported was relatively low (24-h average 63.70 μg/m^3^), which means that even if the population of eMbalenhle was exposed to that average concentration of PM_10_, negative health impacts would be unlikely, as concentrations were below the 24-hour average NAAQS of 120 μg/m3, although some individuals may still be sensitive to relatively low PM_10_ concentrations [8].

**Figure 3:**
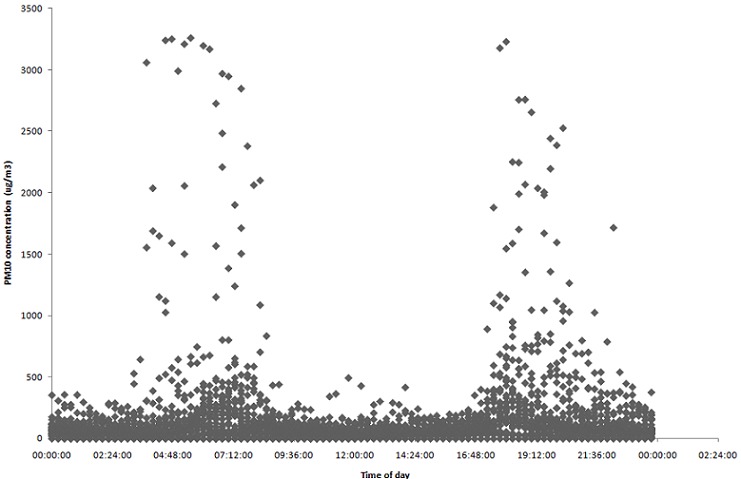
PM10 concentration versus time of day for August 2010 (winter)

Figure 3 presents the concentrations of PM_10_ versus time of the day. High concentrations of PM_10_ were reported in eMbalenhle between 04:45 and 07:45 probably due to the fact that people were burning domestic fuel to prepare for work and they also travelled from different areas during these times, thus elevated vehicle emission levels were present. Figure 3 reflects that between 9:45 and 16:45, the PM_10_ concentrations decreased because most residents were at work, thus the need for energy for cooking and space heating decreased. Between 17:45 and 21:45, the PM_10_ concentrations increased because residents were home and engaged in cooking and household activities requiring domestic fuel use.

A comparison between the winter and summer data from the TOPAS PM_10_ data and DEA PM_10_ data was made (Figure 4a and Figure 4b respectively). Although the monitoring equipment were placed at the same site, monitoring the same pollutant (PM_10_) and within the same period (August 2010 and February 2011), the PM_10_ concentrations monitored with the TOPAS monitor were higher (but following a similar pattern in most cases) than those monitored by the DEA monitoring station. The reason could be that an error occurred during sampling or that one of the instruments was not calibrated appropriately. Generally, when the PM_10_ concentrations monitored with the TOPAS monitor increased, the PM_10_ concentrations monitored by DEA monitoring station also increased and vice-versa. However, the DEA PM_10_ instrument measured on average three times lower than the TOPAS instrument. Hence, to determine the worst-case scenario risk estimates, the TOPAS data were used for the HHRA.

**Figure 4(a):**
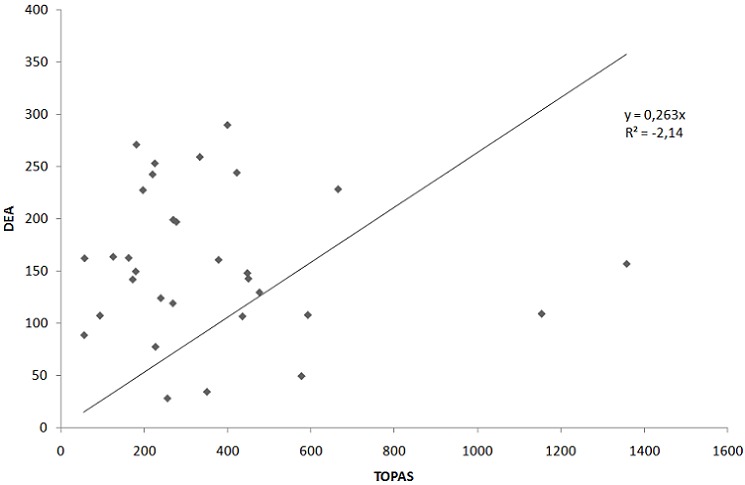
Correlation of the 24-hour PM10 averages monitored with the TOPAS monitor and by the DEA monitoring station in winter

**Figure 4(b):**
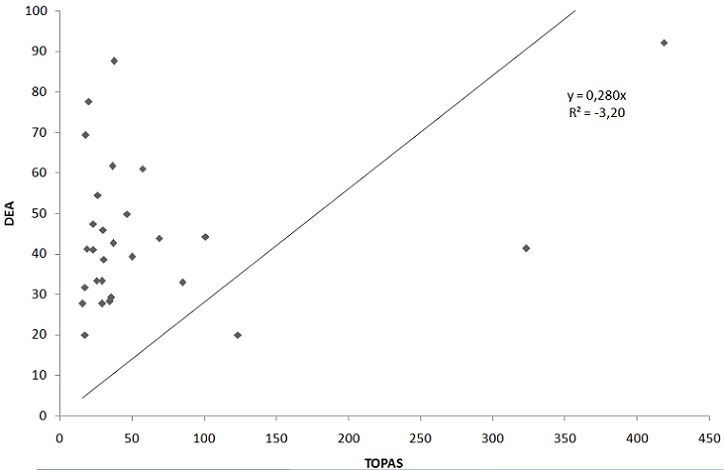
Correlation of the 24-hour PM10 averages monitored with the TOPAS monitor and by the DEA monitoring station in summer

### Human health risk assessment in eMbalenhle

The PM_10_ concentrations monitored in eMbalenhle in August 2010 and February 2011 were applied in the USEPA HHRA model to assess and characterise the potential health risks posed by the community. The population of eMbalenhle was categorised into three life-stage scenarios (infant, child and adult). The three life-stage scenarios were categorised based on the fact that infants, children and adults are exposed differently to PM_10_.

As per the USEPA Exposure Factors Handbook [7], the identified life-stage scenarios have 95^th^ percentile inhalation rates of 7.1 m3/day, 16.6 m^3^/day and 21.4 m^3^/day for an infant (birth to 1 year), a child (6 to 11 years) and an adult (31 to 41 years) respectively. It was assumed that the BW of an infant is 9.2kg, that of a child 52.5kg and that of an adult 118kg [7].

Table 1 gives the SADD of the three life-stage scenarios. The SADD was calculated using equation 2 (multiplying the “safe” PM_10_concentration (C) of 120 μg/m3 with the Inhalation Rate (IR), the Exposure Frequency (EF) and the Exposure Duration (ED), then divided by the Body Weight (BW) multiplied by the Average Time (AT).

**Table 1:** Safe average daily doses of the different life-stage scenarios

Life- stage Scenarios	Safe Average Daily Dose (μg/kg/day)
Infant	0.09
Child	0.03
Adult	0.02

The FADD was calculated using Equation 1 (multiplying the measured PM_10_ concentrations monitored in winter and summer by the IR, the EF and the ED, divided by the BW multiplied by the AT). The results of the FADD for the PM_10_ concentrations measured in winter for the different life-stages are given in Table 2.

**Table 2:** Field average daily doses of the different life-stage scenarios (winter)

Life-age Scenarios	Field Average Daily Dose (μg/kg/day)
Infant	0.28
Child	0.11
Adult	0.06

The results of the FADD for the PM_10_ concentrations measured in summer for the different life-stages are given in Table 3. For each life-stage, the winter dose was higher than the summer dose.

**Table 3:** Field average daily doses of the different life-stages scenarios (summer)

Life-ages	Field Average Daily Dose(μg/kg/day)
Infant	0.05
Child	0.02
Adult	0.01

In order to characterise the health risks posed by exposure to PM_10_ in the different life-stage scenarios, the Hazard Quotient (HQ) was calculated by dividing the calculated FADD by the calculated SADD.

Another method of calculating an HQ to characterise the risk, is to directly divide the actual level of exposure (the concentration measured in the field), by the level of exposure that is not expected to cause adverse effects (the bench mark concentration, which in this case was the national 24-h standard for PM_10_). This method can only be used in the case of inhalation, where both the level of exposure and the benchmark value are presented as a concentration (mass per volume), to have a unit less HQ. The results of the HQ calculations are presented in Table 4 and Table 5. In winter (August 2010), the HQ was 2.97, indicating a moderate risk according to [9] and in summer (February 2011) the HQ was 0.53, indicating that adverse health effects were unlikely, thus a low risk.

**Table 4:** The results of the calculated hazard quotients (FADD/SADD)

Life-stage Scenario	HQ Winter	HQ Summer
Infant	2.97	0.53
Child	2.97	0.53
Adult	2.97	0.53

**Table 5:** The results of the calculated hazard quotients (Field Concentration/NAAQS)

Life-stage Scenario	HQ Winter	HQ Summer
Infant	2.97	0.53
Child	2.97	0.53
Adult	2.97	0.53

The calculated HQs using the IRIS formula, of dividing the concentration by a benchmark concentration, were between 1.1 and 10 for the winter period, which means that the population of eMbalenhle were at moderate risk of negative health effects from exposure to PM_10_. In summer, the HQs were above 0.1 but below 1, which means that the population of eMbalenhle were at low risk and it is unlikely that they may have experienced negative health effects due to PM_10_ exposure.

## Discussion

This study aimed to determine the human health risks posed by ambient PM_10_ concentrations to residents of eMbalenhle in an air pollution hotspot area in South Africa. Similar studies have been carried out elsewhere in the world [10–11]. Our results showed that ambient PM_10_ concentrations were higher during the winter month than the summer month. Possible reasons for the elevated levels in winter may be adverse climatic conditions (such as inversion layers), increased domestic coal burning for space heating, as well as wind-blown dust from mine ash dumps and unpaved roads during the windy month of August. In summer, PM_10_ concentrations were lower, despite industrial emissions that probably remained constant throughout the year, likely due to rain that washed the particulates out of the atmosphere and a decrease in domestic fuel burning due to warmer ambient temperatures as well as less wind-blown dust and the absence of inversion layers.

The PM_10_ concentrations monitored in winter exceeded the national 24-hr average standard of 120 μg/m3 on at least 70% of the days in August 2010. Winter concentrations were the highest between 04:45 and 07:45 and again between 17:45 and 21:45, the times when people would cook and heat their houses against the cold in the evenings, indicating that domestic fuel burning, in this case mostly coal, has an impact on the PM_10_ levels. Even if most households are electrified in eMbalenhle, residents may still prefer to use domestic fuel, at least for space heating.

Several studies have considered the health impact of air pollution on children and infants [12–14]. Children in eMbalenhle may be more exposed to ambient PM_10_ concentrations than infants and adults, because they spend more time outdoors by walking to school, playing outside during school breaks and have sport activities in the afternoons [15]. Infants will be mostly indoors. However, if there are indoor sources of PM_10_ such as domestic fuel use, these infants may be exposed to even higher concentrations than outdoors. The adults living in eMbalenhle may have different exposure levels, depending on their occupation and age. Caregivers and the elderly may be exposed to high levels of PM_10_ if they mostly stay indoors and use domestic fuel. The exposure levels of workers may differ depending on their occupation and whether they work indoors or outdoors. [16] showed that outdoor air pollution in urban areas in South Africa was estimated to cause 3.7% of the national mortality from cardiopulmonary disease and 5.1% of mortality attributable to cancers of the trachea, bronchus and lung in adults aged 30 years and older, and 1.1% of mortality in children under 5 years of age.

The results of the human health risk assessment showed that the infant had the highest calculated dose per kilogram body weight, followed by the child and then the adult, yet the risk was the same for all life stages. The reason for the infant's higher dose compared to the other life stages was due to the breathing rate relative to body weight being greater in infants than in adults. For example, the volume of air passing through the lungs of a resting infant is twice that of a resting adult per unit body weight, which implies that twice the amount of chemical may be taken up by the infant than by the adult under identical exposures. The breathing rate in both boys and girls decreases steadily as the child grows older [17]. A study [11] showed that exposure to even moderate levels of air pollution was associated with increased respiratory symptoms in healthy infants. Ambient air quality standards for pollutants do not distinguish between different life stages, in other words there are not different benchmark concentrations for the different life stages, although air quality standards are supposed to protect even sensitive individuals.

It is envisaged that children who are exposed to pollutants that may have developmental effects or are mutagenic, will be especially susceptible to exposure to such pollutants. The mechanism of action of a pollutant in the human body is therefore important but unfortunately not known for most pollutants. The USEPA therefore suggests that in cases where children are more affected by a pollutant than an adult, an adjustment is needed. In such cases, a 10 fold adjustment is recommended for exposure before 2 years of age, a 3 fold adjustment for exposure between 2 and 16 years of age and no adjustment after age 16 years [18]. This adjustment was not applied in this study since the chemical composition of the PM_10_ was not determined.

## Conclusion

The study found that even though eMbalenhle is surrounded by industries and other sources of PM_10_, emissions at ground level from domestic fuel burning, may contribute mostly to the levels of particulate pollution in eMbalenhle, especially in winter. The results of the HHRA indicated that the infant, child and adult are at equal risk from exposed to the same levels of PM_10_ for the same duration and individuals are more at risk during winter than summer. More studies should be conducted to assess the indoor exposure to air pollution focussing on the more vulnerable groups such as infants, the elderly and those suffering from other respiratory and cardiovascular diseases.
